# Sperm Selection for ICSI: Do We Have a Winner?

**DOI:** 10.3390/cells10123566

**Published:** 2021-12-17

**Authors:** Domenico Baldini, Daniele Ferri, Giorgio Maria Baldini, Dario Lot, Assunta Catino, Damiano Vizziello, Giovanni Vizziello

**Affiliations:** 1 MOMO Fertilife–IVF Center, 76011 Bisceglie, Italy; danieleferrimomo@gmail.com (D.F.); gbaldini97@gmail.com (G.M.B.); dario_lot92@libero.it (D.L.); giovanni.vizziello@alice.it (G.V.); 2 Department of Biology, University of Bari, 70125 Bari, Italy; sissicatino04@gmail.com; 3 Department of Urology, Policlinico San Donato Milano, 20100 Milan, Italy; damiano.Vizziello@gmail.com

**Keywords:** assisted reproductive technology (ART), in vitro fertilization (IVF), intracytoplasmic sperm injection (ICSI), sperm selection, density gradient (DGC), swim-up (SU), horizontal sperm migration, microfluidic

## Abstract

In assisted reproductive technology (ART), the aim of sperm cells’ preparation is to select competent spermatozoa with the highest fertilization potential and in this context, the intracytoplasmic sperm injection (ICSI) represents the most applied technique for fertilization. This makes the process of identifying the perfect spermatozoa extremely important. A number of methods have now been developed to mimic some of the natural selection processes that exist in the female reproductive tract. Although many studies have been conducted to identify the election technique, many doubts and disagreements still remain. In this review, we will discuss all the sperm cell selection techniques currently available for ICSI, starting from the most basic methodologies and continuing with those techniques suitable for sperm cells with reduced motility. Furthermore, different techniques that exploit some sperm membrane characteristics and the most advanced strategy for sperm selection based on microfluidics, will be examined. Finally, a new sperm selection method based on a micro swim-up directly on the ICSI dish will be analyzed. Eventually, advantages and disadvantages of each technique will be debated, trying to draw reasonable conclusions on their efficacy in order to establish the gold standard method.

## 1. Introduction

Intracytoplasmic sperm injection (ICSI) is the method that has definitely revolutionized the field of ART since normal fertilization and ongoing pregnancies can be achieved even with low quality sperm samples and affected spermatozoa. By injecting a single sperm cell in the oocyte, the technique bypasses several biological barriers that naturally select the gametes to achieve optimal embryonic and fetal development.

ICSI has been proven to show the same efficiencies as in vitro fertilization (IVF), a simpler technique where the oocytes, after pick-up, are left with the prepared sperm sample overnight and fertilization occurs following a sort of natural sperm selection [1]. Nowadays, ICSI is the most used technique infertility treatments in developed countries, replacing IVF as the first choice [2,3].

In fact, since ICSI allows the oocytes to be fertilized by sperm cells that may not be competent, concerns about male inheritance patterns are growing [4].

It has been estimated that about 50% of couples with fertility issues are affected by low sperm cell quality [5] and the reproductive outcome is generally influenced by the paternal genome condition. Some authors [6] suggest that paternal age might impact the quality of the seminal fluid.

This is widely confirmed by many studies based on animal models: when exposed to low doses of chemotherapeutic agents providing an amount of reactive oxygen species (ROS), animals produce sperm cells with epigenetic defects, which lead to deformity in the offspring and are often inherited in the following generations [7]. Human epidemiological studies [8] have shown a correlation between paternal age and correct neurological development. Some studies have shown that the lack of acrosomal reaction and the persistence of the perinuclear theca on the sperm cell head is associated with a delay in de-condensation of the genetic material at the time of fertilization [9]. Other studies have shown that male subjects born from ICSI generate seminal fluid with a low number of cells and with reduced motility when compared to subjects born spontaneously [10]. Further, ICSI has been associated with an increased risk for many health issues, ranging from premature births and diverse metabolic disorders in the offspring to more severe complications, such as abortions, congenital malformations, and imprinting disorders [11]. It has been suggested that one of the reasons for the relatively low efficiency of ART is that we currently lack an effective methodology to separate this specific sperm subpopulation for its use in ARTs [12,13].

In this perspective, effort to identify the eligible technique to select competent sperm cells for ICSI seems mandatory.

## 2. Natural Sperm Selection

In nature, the process that leads to the selection of the best sperm cells, capable of fertilizing the oocyte in the female genital tract, is very selective and it involves high morphologic qualities and dynamic features. Leaving the seminal plasma in the vagina, sperm cells perform the first step that leads to this process.

Only a small portion of the sperm cells that swim from the uterus to the oviduct can be collected in niches, where cells interact with unknown receptors to form a sperm reserve, as in some animal species the sperm cells can stay in the fallopian tubes even for months before ovulation. However, in the human species, the fertilization window is reduced, and the sperm cells can survive no longer than 5/6 days [14]. This process allows the sperm to reach complete capacitation, which is based on plasma membrane cholesterol ultrastructure changes; it leads to an increase in concentration of intracellular ions, such as Ca^2+^, that switch the motility patterns of tails to hyper-activation. Moreover, phosphorylation on tyrosine residues determines the preparation for the acrosomal reaction event [15]; subsequent interactions with molecules, such as secreted protein or hormones, from the female reproductive tract, modulate the swim, by a chemotactic and thermotactic effect, towards the oocyte.

Most chemoattractants are still unknown, but progesterone released from cumulus cells certainly plays a key role, allowing the interaction with oocyte receptors and preparing for acrosome reaction [16].

One of the main goals of artificial reproductive technology (ART) is to simulate the strict selection process that takes place in the female tract to improve the reproductive outcome.

Unfortunately, classic methods have given unsatisfactory results, as they rely on separation mechanisms dependent on morphology and motility, such as swim up (SU) and density gradient centrifugation (DGC) [17]. To be adopted in the ART routine procedures, sperm cell preparation techniques should be simple, cheap and fast, allowing a highly efficient selection that differentiates motile and morphologically normal spermatozoa from other cell species, leukocytes or bacteria and toxic substances, avoiding the production of ROS [18].

All current methodologies improve sperm cells quality, but none have been associated with a significant increase in clinical results [17]. Methods for sperm cells’ selection are categorized into classic techniques based on sperm motility or density, and advanced methods that rely on membrane surface charge, high-resolution morphology, and nuclear or membrane integrity. Nowadays, the most used techniques in the ART lab are SU and DGC [18]. Here below the Table 1 represent a summary of all the sperm cell preparation techniques described in the review with advantages and disadvantages of each of them. 

## 3. Classic Methods

### 3.1. Swim-Up

Lopata and Patullo [19] first described in 1984 the basic principle of recovery motile spermatozoa that manage to migrate through a medium placed on top of the seminal plasma.

**Figure 1:**
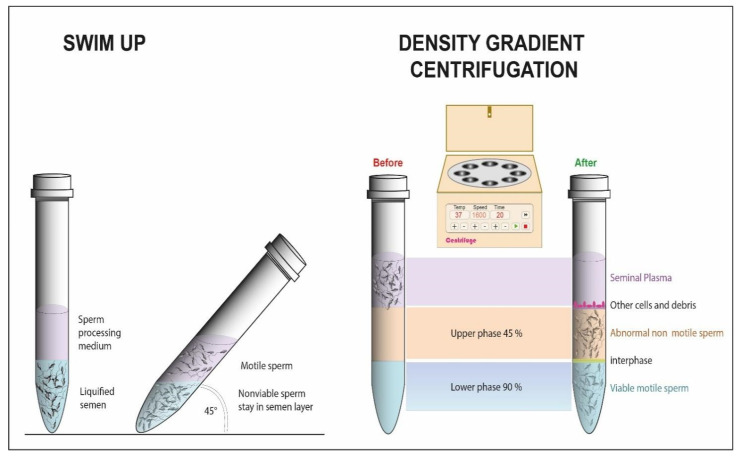
Schematic representation of swim-up and density gradient centrifugation.

The swim-up (Figure 1) is known as the most elementary method for sperm cells’ preparation. The sample is centrifuged with a medium consisting of polysiloxane microparticles to remove the seminal serum and subsequently left for 60 min in an incubator at 37 °C, with a 45° inclination and an overlapped layer of medium. The principle of the technique is based on the attraction of sperm cells to the most enriched area of the liquid. These conditions should promote capacitation and migration: cells with progressive motility can rise from the pellet through the medium and they can be recovered from the surface [64]. There are also several variations of the technique that avoid the centrifugation process where the sperm cells can pass directly from the seminal fluid to the media surface, which is simply placed over the sample [23].

This method is applied both for in vitro fertilization (IVF) and ICSI, and is recommended when motile spermatozoa in the sample are in low percentage [23]. Although there is centrifugation, swim-up is a very soft technique, it produces a small load of ROS, but it recruits only mature activated sperm cells [20]. On the other hand, swim-up has some drawbacks. In the case of samples with high volume or elevated concentration, the method requires a distribution between several tubes of the sample to emphasize the area of contact to the culture medium; this subdivision leads to a burden of workload. Another weak point is the small portion of spermatic cells retrieved; only a maximum of 10% [24] of the total sperm cells population becomes available for further use. If samples are not centrifuged gently, many motile cells from pellets could potentially become anchored to the bottom of the tube, and never reach the culture media layer.

### 3.2. DGC

In DGC [25] (Figure 1), sperm cells are placed over a continuous or discontinuous density gradient and then centrifuged. Separation occurs as a function of density and motility; the fastest spermatozoa will migrate to the bottom of the tube.

When left on top of the solution with a higher density, the sample is centrifuged for about half an hour. All sperm cells pass through gradients, but motile spermatozoa swim vigorously instead of remaining subjected to the kinetics of centrifugation. Quicker sperm cells form a silky pellet at the bottom of the tube while immotile cells and debris remain between gradients.

The substance used to constitute the gradients should not be toxic for spermatozoa and stable in solution, to avoid any pH alteration or osmolarity.

More specifically, we find hydrophilic organic molecules, such as sucrose or sucrose copolymers (Ficoll) [26], which are less toxic than mixed compounds, such as colloidal silica coated with polyvinylpyrrolidone (Percoll), producing reduced negative effects on the ultra-sperm structure [27]. This method allows the isolation of motile spermatozoa even from a sample with low sperm motility. However, morphology and motility parameters vary considerably among different studies in the literature. The reason for this bias may be caused by different initial conditions, such as concentration, volume, number of layers, g-force applied and centrifugation time. Furthermore, the technique takes longer. DGC is low-yielding when the viscosity of semen is altered, and it produces a higher amount of ROS due to mechanical stress caused by centrifugation. When samples are excessively concentrated, clusters of different types of cells prevent movements through gradients. Unfortunately, density gradient centrifugation is even more expensive than the classic swim-up [24].

Several studies have tried to clarify which one is the ideal method for sperm cells preparation, inspecting morphological and dynamical parameters. Nevertheless, the results available in the literature to date are contradictory.

Since the studies published during the 1990s [65], it has been demonstrated that the SU method selects sperm cells with better motility, vitality and morphology than DGC. Moreover, the latter is less efficient than SU in selecting sperm cells with intact DNA [66]. While these theories have been reinforced by more recent studies [67], in contrast, other authors have shown that DGC is better than SU to select a higher percentage of capacitated sperm [68] with hyper-activated motion even in cases of subfertility [69]. Other scientists suggest that neither method provides a cell population with acceptable levels of intact DNA. Centrifugations related to both techniques might produce an increase of ROS harmful for the spermatic nuclear material [70]. Both methodologies are regularly exploited in the ART lab. Unfortunately, by relying only on motility, these techniques cannot be associated with an optimal quality of spermatozoa [71]. Further studies [21] have shown that by using both methods in combination, better results can be obtained in separating sperm cells with damaged DNA, removing them from the rest of the sample [22]. It has also been observed that spermatozoa with less nuclear fragmentation are correlated with an improvement of the embryo quality and a reduced probability of miscarriage [72]. In performing ICSI, it is crucial to choose sperm cells with optimal characteristics, and using these combined methods, a higher fertilization rate can be achieved even in cases where patients have a normal spermatozoa density but reduced motility.

## 4. Advanced Methods

### 4.1. Selection Methods for Sperm Cells with Reduced Motility

When the sperm sample is retrieved by testicular aspiration (TESE) [73] or epididymal aspiration (MESA) [74], the spermatozoa might appear immotile due to the lack of complete maturation that takes place in the final tract of the epididymis [75].

It is important to underline that in these cases, the SU and DGC techniques, that exploit dynamic characteristics for separation, are inadequate.

Marques De Oliveira [76] proposed the technique of mechanical touch (MTT) for the selection of immotile spermatozoa as potentially suitable for ICSI. Briefly, if the tail is still flexible and recovers its original position being touched by an ICSI injection needle, it is considered viable. Instead, rigidity and incapacity to recover the initial tail position is thought as a sign of non-viability [77]. The success and reliability of this technique depends mainly on the expertise of the biologist, and this could be considered as the only current drawback of the latter [76].

**Figure 2:**
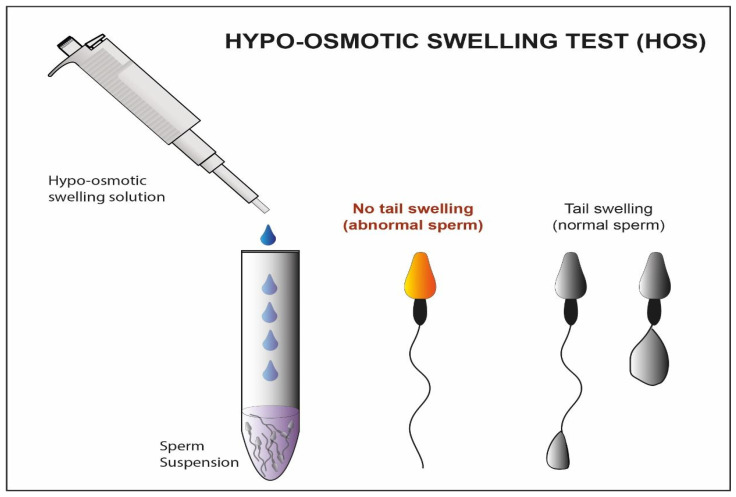
Schematic representation Hypo-osmotic swelling Test. In this figure, we observe the difference between the abnormal sperm with not swelled tail and the normal sperm with swelled tail after treatment with a hypo-osmotic solution.

An alternative selection technique is the hypo-osmotic swelling test (HOST) [28]. This method (Figure 2) assumes that the tails of viable spermatozoa swell and bend if they are introduced into a hypoosmotic environment, due to the activity of the osmo-sensitive calcium membrane channels [78,79]. HOST can be used to estimate the percentage of integrity in chromatin. Seven distinct patterns of swelling are related to different levels of chromatin integrity. This allows the recognition, through microscopy, of sperm cells with better nuclear material for ICSI treatments [29,30]. According to the World Health Organization laboratory manual for the examination and processing of human semen [23], HOST is advised in cases of asthenozoospermia to collect suitable spermatozoa for ICSI. Some studies suggested that HOST should be employed in a subsequent ICSI cycle following total fertilization failure [80].

Some controversial studies instead suggest that the use of the HOST is associated with a low fertilization rate, and non-viability of sperm after 30 min of incubation in hypo-osmotic solution [31]. Moreover, biologists must work with extremely limited quantities of testicular sperm in many cases, and the HOST test involves dilution of the sample.

Hence, this method should be prerogative depending on the specific ART laboratory settings and on the amount of sperm sample available [32].

**Figure 3:**
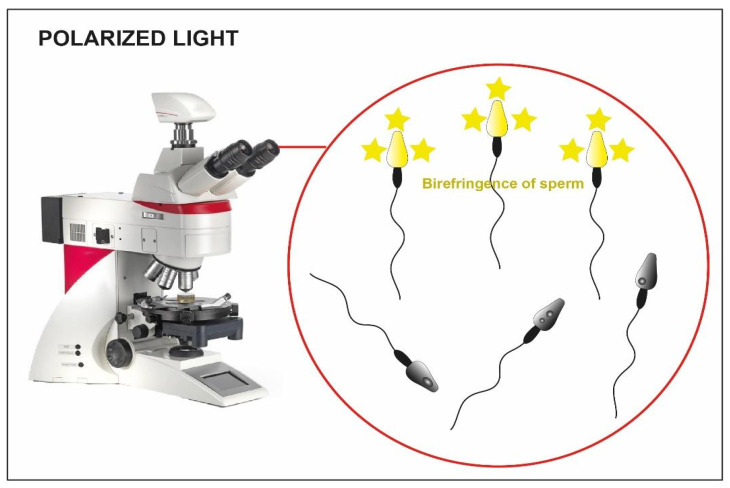
Microscope implemented with polarized light. The birefringence of the heads is clear in the viable sperm (yellow heads) compared to the not viable one where the birefringence is absent (dark heads).

Using polarized light implemented in the microscope (Figure 3) is another possible strategy for the selection of immotile sperm cells [33]. Some research teams have proposed its use to verify the birefringence of sperm cell head as an index of suitability. As a matter of fact, the sub-acrosomal protein filaments extend themselves longitudinally, giving a typical pattern of birefringence to the sperm cell head [34]. In some published studies, the selection of non-motile spermatozoa with birefringent heads resulted in an increase in clinical pregnancy and implantation rate (58% vs. 9% and 42% vs. 12%), compared to a control group with immotile spermatozoa (where polarized light was not used) [81], and an increased implantation rate when the HOST technique was used instead as selection method (45% vs. 11%) [82].

Unfortunately, even this technique presents some disadvantages: the polarized light implemented in the microscope is expensive and there is a lack of data regarding the integrity of the sperm DNA. Furthermore, the success of the latter is based largely on the experience of the operator.

In this perspective, it is fundamental to underline that sperm DNA integrity is essential for fertilization and early stages of embryo development [83]. It is known that sperm cells are not able to repair the DNA after spermiogenesis since they do not express a DNA repair mechanism. [84,85]. Due to the sperm physiology and the environment that they are exposed to, DNA damage is inevitable, but post-fertilization it can be repaired at least partially. When the damage exceeds the oocyte’s repair capacity, it might result in absence of fertilization [86]. Studies have shown that the chance of both natural and artificial conceptions is reduced if the DNA fragmentation index is more than 30% [84,87,88]. Hence, a wide range of techniques have been developed in order to assess the sperm chromatin and DNA integrity, despite standardization or guidelines to uniformize results remain absent [89].

**Figure 4:**
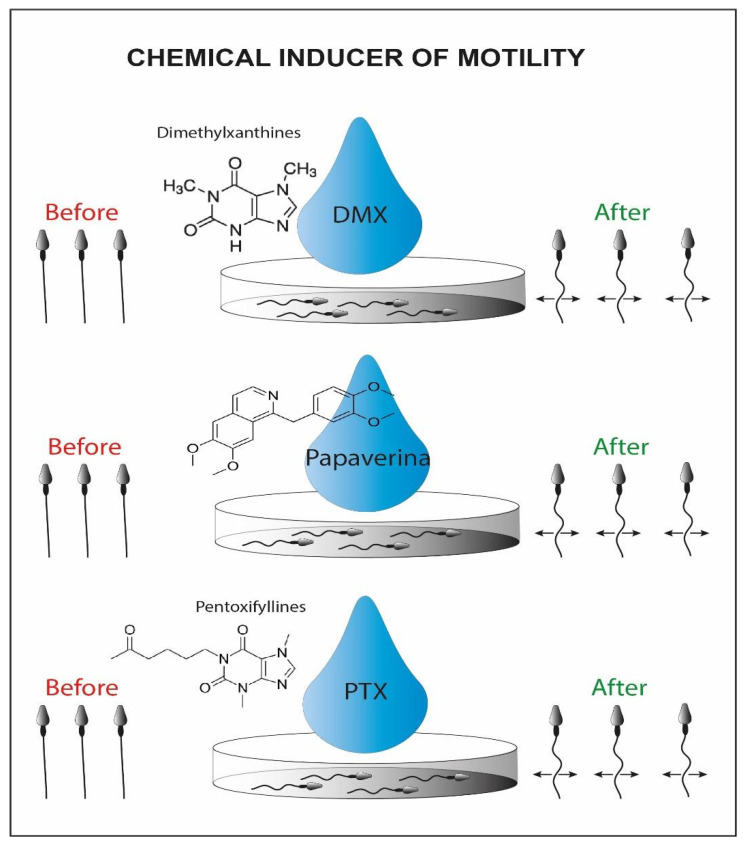
Schematic representation of chemical inducers of motility. On the left we can recognize the initial immotile sperms and on the right side the viable motile sperms activated by the chemical inducer.

A group of researchers has shown another potential method to recover immotile but suitable sperms for ICSI. It exploits the chemical inducers of motility (Figure 4), that belong to the class of phosphodiesterase inhibitors, such as pentoxifylline (PTF), dimethylxanthines and papaverines [90], allowing the motility reactivation of spermatozoa recovered from the testicular and epididymal level [91]. Using this motile fraction, it was possible to obtain fertilization, normal pregnancies, and an increase in birth rate through ICSI [92,93,94]. Some doubts about the technique concern the intrinsic toxicity of these molecules towards the genetic material, but results from studies suggest that in vitro treatment adequately improves motility of immotile sperm, without leading to acrosome reaction, DNA damage, and viability loss [95,96]. Comparing this technique with HOST, researchers have obtained a higher fertilization and pregnancy rate (32% vs. 16%) [97].

**Figure 5:**
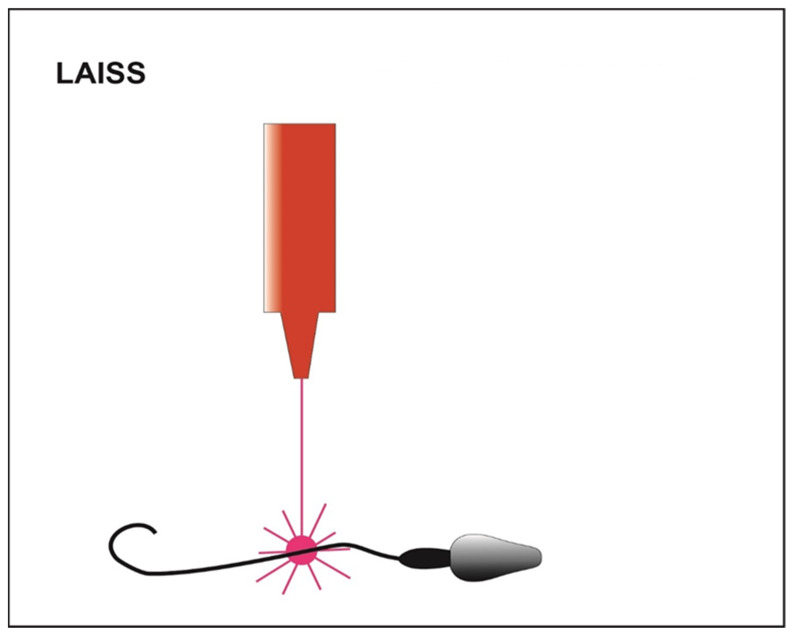
Schematic representation of LAISS (laser-assisted immotile sperm selection). The laser irradiation generates a slight movement of the tail in those viable sperms initially immotile.

Alternatively, motility can be further reactivated by using a laser incorporated in the magnification system that is shot at the immotile sperm cell tail (LAISS laser-assisted immotile sperm selection) (Figure 5) [35]. Laser irradiation causes the release in cytosol of second messengers, such as Ca^2+^ or ROS, and an increase in the synthesis of ATP, which could generate a slight movement of the tail [98]. The sperm cell is then considered viable when its tail is coiled after the laser shot [36]. On the other hand, some authors claim that high laser doses cause an excess of potentially toxic ROS [39]. Furthermore, more Ca^2+^ influx induces the hyperactivity of Ca^2+^-ATPase calcium channels and exhausts the ATP reserves of the cell. This process could lead to depletion of cell channels activity, correlated with an increase in internal osmotic pressure causing the swelling of the sperm cell and subsequently the rupture of its plasma membrane [40]. Contrary, other authors suggest that this technique does not damage the spermatic membrane and does not affect the percentage of fragmentation of the genetic material [99]. LAISS is also an alternative to the chemicals’ motility activators, which could be potentially toxic [100]. In addition, it significantly increases the embryonic segmentation and post ICSI birth rate, using testicular or ejaculated spermatozoa, when compared to control groups [101]. LAISS is the recommended technique in case patients suffer from primary ciliary dyskinesia [37] or Kartagener’s syndrome [38]. Despite the high potential of the method, the complexity and cost are the main reasons why it is not adopted in routine practice in ART laboratories.

**Figure 6:**
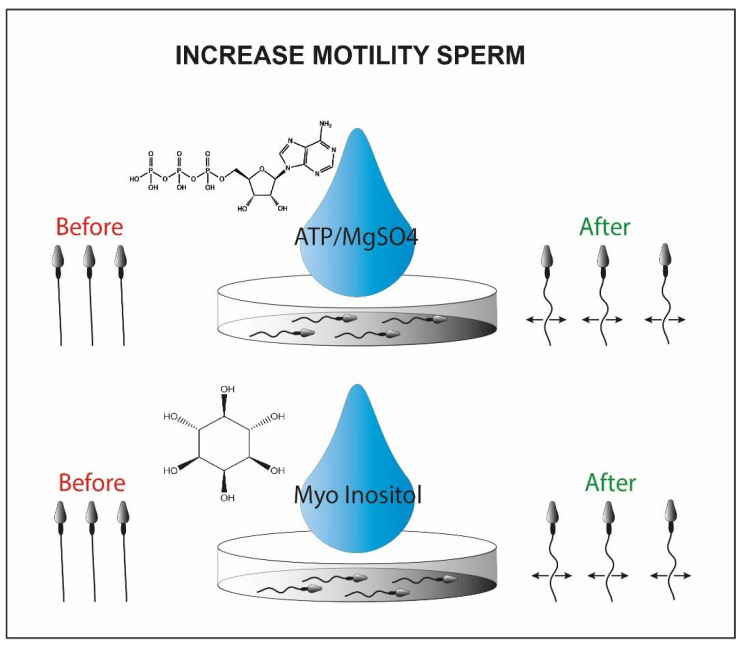
Sperm motility enhanced by ATP/MgSo4 or myo-inositol. Briefly in the picture we observe the immotile sperm before the treatment with the chemical inducer (left side) and the motile sperm cells after the chemical exposure (right side).

It has also been demonstrated that the use of ATP/MgSO4 generates an increase in motility in testicular seminal samples, generating a weak contraction of the flagellum (Figure 6). The results of the study have shown that using ATP as a solute, the induced motility increases significantly, and viable sperm cells can be captured and used for injection through ICSI [102].

Alternatively, the myoinositol can be used to isolate viable sperm cells for ICSI. It represents the most abundant stereoisomer of the inositol class modulating the intracellular concentration of Ca^2+^ [103]. This is synthesized in two steps by two enzymes, myo-1-phosphate synthase and myo-monophosphatase-1, located in high concentration in the testicular mesenchymal tissue [104,105]. By incubating sperm cells frozen and then thawed from oligoasthenospermic patients with myoinositol, some research groups were able to recover a portion of sperm cells with significantly increased motility [101]. Myoinositol generates an increase in mitochondrial membrane potential, resulting in an intracellular increase of Ca^2+^ [106]. The research group performed a series of ICSI by pre-incubating sperm cells from oligoasthenospermic patients in solutions containing myo-inositol [107]. The increase in motility and fertilization rate has generated encouraging results. Unfortunately, one of the key objectives for ART laboratories is to mimic the culture conditions in vivo and myoinositol, as the ATP/MgSO4, could generate an uncontrolled increase in the mitochondrial membrane potential which could lead to the occurrence of toxic effects. Further studies should be conducted to incorporate this practice into clinical routines [108].

### 4.2. Sperm Cells Selection by Membrane Characteristics

The outer sperm membrane is critical for their functionality since it is firstly involved in many aspects of the fertilization such as capacitation, oocyte binding and acrosome reaction [109,110]. In order to select high-quality sperm cells, methods that gain benefits from the characteristics of their membrane have been studied.

The zona pellucida binding assay (ZBA) is a technique that allows the selection of mature and competent spermatozoa. The zona pellucida has specific receptors for the latter [111]. An immature oocyte, recovered from the cohort obtained from a patient’s oocyte retrieval, is incubated with the sperm cells of interest, previously selected with DGC. Sperm cells that bind the zona pellucida are retrieved through a microinjection needle, subsequently used to perform the ICSI. From some studies, it emerges that sperm selection with this method leads to improved embryo quality and an increase in implantation rates [112]. Although there is no routine application in the IVF laboratories, probably due to the heavy workload, the technique is recommended for cases of repeated fertilization failure [113].

**Figure 7:**
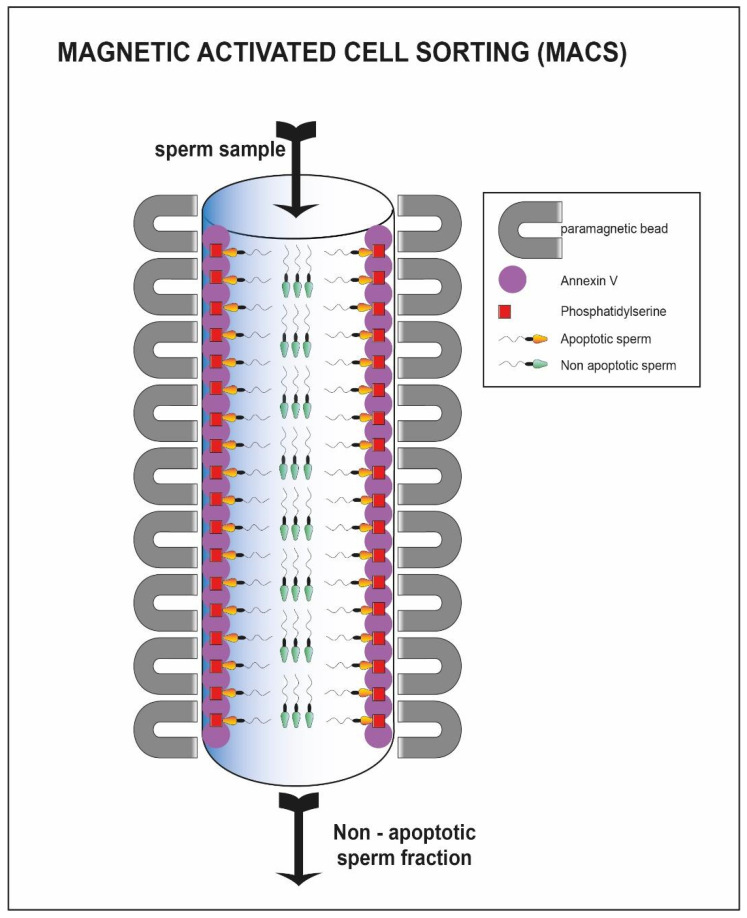
Sperm cell passing through column with annexin V. The sperm cells able to pass through the column represent the viable one (green heads) while the apoptotic fraction remain trapped inside the column (yellow heads).

A further method that takes advantage of the membrane characteristics is the magnetic activated cell sorting (MACS). This methodology allows the selection of the non-apoptotic portion from a sample of interest [114]. It involves the use of magnetic microspheres conjugated to Annexin V (AV-MACS) (Figure 7) [115], that have a high affinity for phosphatidyl-serine. The latter is normally exposed on the outer side of the membrane when the sperm cells are in an apoptotic state [116]. The seminal sample of interest passes through a column containing microspheres, to which the annexin has adhered. The non-viable spermatozoa remain trapped inside the column, while the viable fraction is eluted, improving the vitality characteristics of the starting sample [41,42].

Several studies have shown that the MACS method is efficient in cases of high nuclear fragmentation, idiopathic infertility, and patients with varicocele [43,44]. When this technique was performed in combination with classical procedures, such as swim-up or density gradient centrifugation, the recovered spermatozoa exhibited an even lower percentage of fragmentation [45,117]. A study conducted on a group of oligoasthenzoospermic patients reported that the seminal sample subjected to MACS led to an increase in embryonic segmentation and pregnancy rate when compared to simple centrifugation on a density gradient [118]. Unfortunately, also in this case, the literature remains incomplete on the percentage of live births, and the method does not allow discrimination between the type of motile spermatozoa selected.

**Figure 8:**
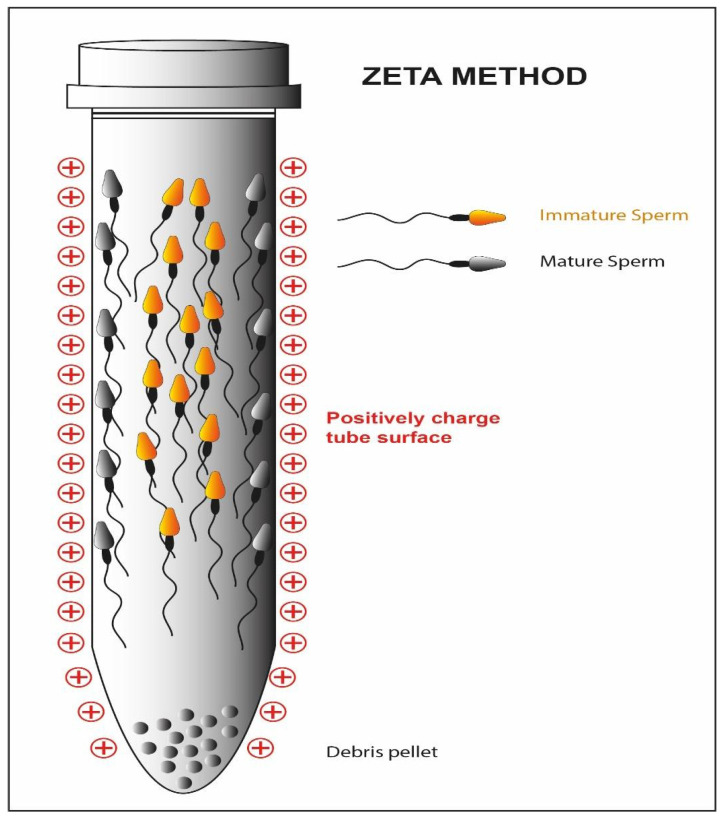
Schematic representation of the Zeta method. The high negative charge of the mature sperm cell membrane reacts with the positive charge of the tube while the immature fraction is detached form the tube.

Hyaluronic acid is the main component of the extracellular matrix surrounding the cumulus-oocyte complex [119]. Only the sperm cells that have successfully completed spermatogenesis and maturation are able to show the polysaccharide-binding receptors on their outer membrane [46,47]. Moreover, they are usually characterized by a normal morphology and a low percentage of fragmentation of the nuclear material [48]. Exploiting these characteristics, a methodology for sperm cell selection has been devised where spermatozoa are incubated in plates or in media containing hyaluronic acid; only the sperm cells able to bind the molecule are then used to perform a modified version of ICSI: physiological intracytoplasmic sperm injection (PICSI) [120,121]. The selection should result in an increased fertilization rate, but in this case, the clinical data are conflicting. Some studies suggest that both the fertilization rate and the percentage of top-quality embryos benefit from the technique (92 vs. 86; 36 vs. 24%) [122], but other research groups do not confirm these improvements [49]. Studies show that a negative charge is present on the sperm cell membrane [123]. The Zeta method (Figure 8) uses this feature to separate sperm cells containing the Y chromosome from those containing the X chromosome [50]. Two distinct research groups have developed two different methodologies, one using a positively charged centrifuge tube [54], the other using migration in an electrophoretic field [55], which allow the collection of live spermatozoa with normal morphology and a high percentage of the integrity of the genetic material [51,52,124,125]. To date, only a randomized study has been conducted on spermatozoa selected with the Zeta method and subjected to ICSI [53]. This showed a significant increase in the percentage of top-quality embryos (45.83 ± 3.11% vs. 35.38 ± 4.64% (*p* = 0.04)) and in the pregnancy rate (39.2 vs. 21.8 (*p* = 0.009) as compared to the method of density gradient centrifugation.

### 4.3. Selection Based on Morphology–IMSI

**Figure 9:**
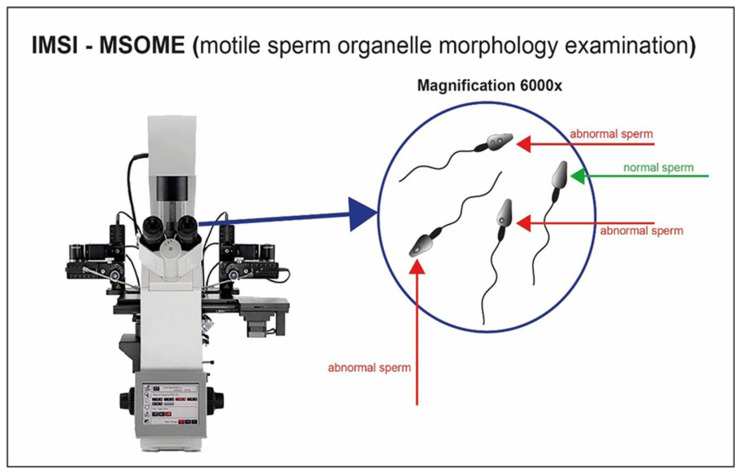
Sperm cell visualization with magnification system 6000×. Sperm cells with appropriate morphology are well selected through the high magnification.

The analysis of semen quality has for a long time been associated with the morphological evaluation of spermatozoa. The introduction of digital microscopy has made its possible to analyze the ultrastructural characteristics of motile spermatozoa (MSOME; motile sperm organelle morphology examination) [126]. The integration of the MSOME into the ICSI method allows a high-magnificence micro-injection (IMSI) (Figure 9) [127]. The selection of motile spermatozoa with few vacuoles and normal nuclear morphology is possible through a 6000× magnification system integrated with the micromanipulation system [128]. Again, the scientific data are promising but conflicting. Using this method, some studies [56] have revealed that the presence of large vacuoles in the sperm nucleus is associated with a high level of nuclear fragmentation. In case of repeated fertilization failure with the ICSI method, the IMSI method has been suggested as preferential [57]. On the other hand, some authors [58] did not find significant differences in terms of fragmentation or pregnancy rates between ICSI and IMSI. Recently, in a study conducted on a large sample of semen analyzed with MSOME, no correlation was found between the presence of vacuoles in the nucleus and DNA fragmentation [59]. The authors argue that vacuoles are physiologically present in the sperm head, and they do not affect their functions. Unfortunately, due to lack of literature, the timing of the analysis and the cost of the equipment, the IMSI method has not been fully adopted in the routine practices of ART clinics.

### 4.4. Dynamic Selection of Spermatozoa

The three main mechanisms that control the movements of spermatozoa within the oviducts, rheotaxis, chemotaxis and thermotaxis, have been exploited as methods for sperm cell selection, to improve the ART outcomes:

1. Human spermatozoa orient their motion, by rheotaxis, against the fluid stream directed towards them [129]. Rheotaxis is defined as the tendency of certain living beings to move in response to the mechanical stimulus of a current of water. Some studies have confirmed the presence of a flow-directed towards the uterus in the oviductal region, capable of increasing its intensity after intercourse, which is able to attract the sperm cells [130]. In the technique developed by Nagata et al. [131], using bull sperm as a mammalian model, a flow is created through a series of microchannels towards a well where the sperm sample is delivered. In response to the flow, the spermatozoa swim towards it passing through the microchannels and finally reaching a receptive well where they can be collected for ART applications. Another study was conducted on normospermic patients [132] to select spermatozoa using rheotaxis: compared to an untreated sample and a sample subjected to density gradient centrifugation, the recovered spermatozoa showed higher chromatin compactness (99% vs. 71% vs. 83%). In recent years, attempts have been made to include the concept of rheotaxis in microfluidics [133], but despite the promising data, an analysis on the quality of selected spermatozoa has not been conducted and the passive nature of rheotaxis correlates to the inability to discern the different types of motility.
2. Progesterone plays the main role as a chemo-attractor for the navigation of spermatozoa through the environment surrounding the cumulus-oocyte complex [134]. Only capacitated spermatozoa possess the receptors to recognize and bind this type of molecule [134]. Some sperm cell selection techniques exploit this feature to distinguish capacitated spermatozoa from non-capacitated ones. Several studies have been conducted on different types of sperm populations, using a device where a progesterone concentration gradient is constituted (sperm selection assay) [135]. In this method, two wells are connected by a 2 mm length per 2.5 mm diameter tube. One of the wells is filled with a media containing the chemoattractant molecule in solution (progesterone for instance) and the other well is filled with the human sperm sample. The chemoattractant diffuses through the tube generating a gradient and the spermatozoa respond by moving towards the higher concentration and accumulating in the initial well free of cells where they can be used for ART applications. The recovered spermatozoa from this technique exhibit better morphology, less DNA fragmentation and a reduced rate of apoptosis as compared to those selected with density gradient centrifugation [136]. Although the results are also promising in this case, further studies need to be conducted to confirm the improvements in the clinical field.
3. The spermatozoa can direct their motion according to the variation in temperature, moving from cold areas to warmer areas [137]. Several studies show that this mechanism underlies the movement of sperm cells from the fallopian tubes to the ampulla [138,139]. Even in this case, however, only the capacitated spermatozoa can respond to the temperature gradient, making this motion a mechanism for the selection of sperm cells with better fertilizing characteristics. The method of sperm selection by thermotaxis was developed by Pérez-Cerezales et al. [140]. Spermatozoa are placed in a drop of medium, connected by a capillary to a second drop free of cells. A temperature gradient is generated between both drops, as the highest temperature is maintained in the drop free of cells. Spermatozoa respond by thermotaxis migrating towards the warmer temperature and accumulating in the second drop where they can be collected for downstream applications.

The purpose was to select spermatozoa with high nuclear integrity, compared to those isolated by swim-up. Unfortunately, even in this case, the literature does not offer a variety of studies that fully confirm the improvements for ART efficacy in the human clinic.

### 4.5. Microfluidics Applied to Sperm Selection

Technologies related to microfluidics are rapidly growing within ART laboratories. Among the first experiments with this methodology, Smith and Takayama [60] have published a series of articles demonstrating the efficiency of the method for the selection of high-quality spermatozoa.

**Figure 10:**
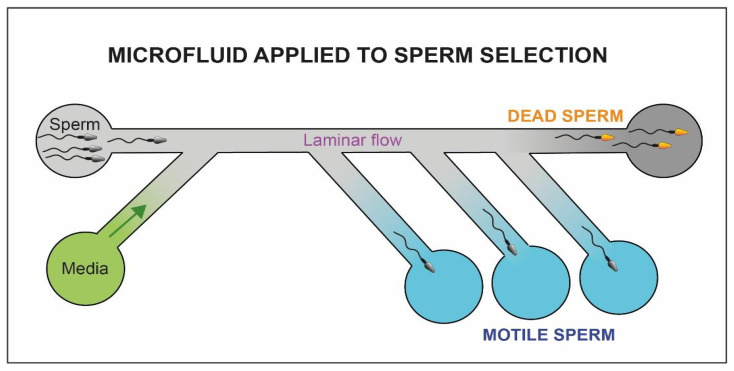
Schematic representation of the microfluidic system. The motile fraction of sperm cells is able to swim through the flow and be collected in separate chambers (blue chambers) while the immotile sperm cells (yellow heads) and debris reach the exit of the microfluidic system (dark chamber).

By controlling fluid dynamics (Figure 10), within millimeter diameter capillaries, it is possible to mimic the physiological conditions of pH and temperature of the female genital tract [141]. Hence, we could potentially select spermatozoa with increased motility through flows [142], chemical gradients [143] or electrophoretic fields [144]. The method of Smith and Takayama [60] uses two parallel laminar flow channels. While the motile spermatozoa can move through the flows and be eluted separately, the debris and immotile cells are passively transported from the entrance to the exit of the capillary canal. Compared to a classic density gradient centrifugation, the morphology and motility of the selected spermatozoa are significantly increased (98% and 22%). Parrella et al. [61] report in a study performed on a small pool of couples undergoing ICSI, that spermatozoa selected using these devices could be linked to an increase in pregnancy rate (71%).

In the near future, microfluidics based on morpho-dynamic characteristics will be replaced by further innovations, including integrating chemo-attracting selection systems [145,146], cumulus cells in the selection chamber or even layers of oviductal cells [147].

The application of optical systems to microfluidics could further improve the selection system [148]. Raman spectroscopy allows the discernment of sperm cells with high nuclear integrity from sperm cells with fragmented DNA [149]. By coupling the three-dimensional imaging to the selected channels, it is possible to accurately identify the different types of flagellar movement using a digital sensor, consisting of a semi-conductive material, which can virtually reconstruct the cell volume by recording the differences in the types of interference between a reference light wave and a scatter from the sample [150]. Using this method, De Wagenaar B. et al., were able to trace the profile of the flagellar beat of hyper-activated spermatozoa [151].

Among the advantages that microfluidics certainly offer are automation, scalability and reduction in diagnosis and preparation times. Unfortunately, the high cost of instrumentation and poor yield of the sample in terms of volume represent the main limitations of the method.

### 4.6. Horizontal Sperm Migration

**Figure 11:**
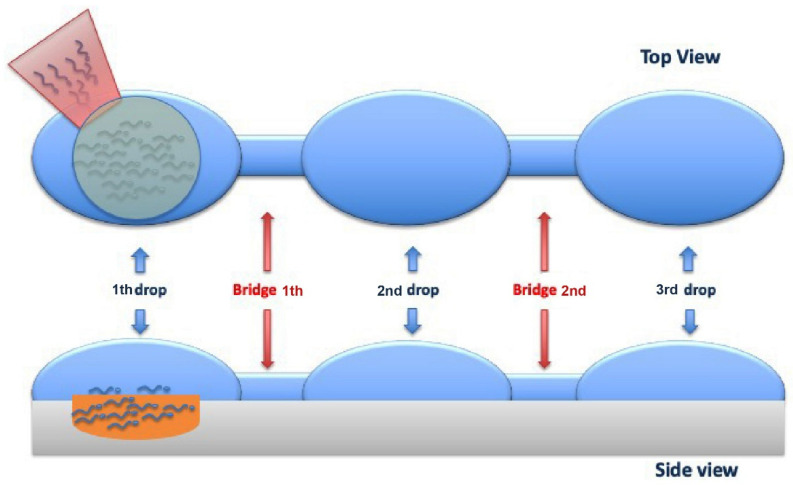
Top and Side viewing of the sperm cells horizontal migration from the first drop (where the cells are added) to the third drop (where the sperm cells are aspirated) through 2 bridges that link them.

The method developed by our research group [62] called “horizontal sperm migration” involves the preparation of a modified ICSI plate, including three additional drops of a solution containing HEPES (G-mops^®^ Vitrolife, Göteborg, Sweden). The drops are connected through a medium bridge shaped by swiping the denuding pipette across them. In relation to concentration and motility, observed from an analysis previously performed under the microscope, 1 to 5 µL of ejaculate are loaded into the proximal portion of the medium strip, about ten minutes before performing the oocyte injection. During ICSI, an adequate number of spermatozoa reaches the distal edge of the furthest drop; some of these can be recovered from the injection needle, to be moved to the PVP and carefully selected for fertilization (Figure 11).

The technique allows the recovery of spermatozoa with high motility, normal morphology and minimal damage to the DNA, using a fast, safe, and economical procedure. Comparing the clinical results obtained from this new selection method with the classic swim-up technique, no significant differences were found in terms of fertilization and implantation rate. On the other hand, segmentation and blastocyst rates are higher in the horizontal swim-up, suggesting that this process that generates a lower quantity of harmful oxygen reactants is correlated with better seminal quality and a lower DNA fragmentation. In line with our observations, clinical and ongoing pregnancy rates are numerically better in the horizontal swim-up than in conventional methods although there is no significant statistical difference. By comparing a variant of horizontal migration with density gradient centrifugation, a previous study [63] found out that fertilization rates between the two methodologies are similar, but blastocyst and pregnancy rates results improved in favor of the innovative technology.

In terms of timing, while for the swim-up and the centrifugation on the density gradient it takes between 50 and 80 min for the preparation of samples, with this innovative method it is possible to perform the oocyte injection 10 minutes after placing the migrating semen sample. Furthermore, the procedure is much less expensive since there is no necessity to use culture media required for preparation in other methods. Moreover, by avoiding the use of several tubes of media, the absence of bacterial contamination is also ensured. Furthermore, it is also possible to improve the recognition system and avoid mismatch errors in patient identification.

## 5. Conclusions

Each method has shown advantages and disadvantages, but none seem to be superior at this time (Table 1). Despite the countless number of methods, none have shown outstanding quality or results in increasing pregnancy rates. To date, there are many new methods that present encouraging results, but we still do not have a randomized controlled trial with sufficient data to demonstrate that one of these could be eligible as a gold standard technique. Considering this, all the methods listed in this paper offer applicability in different contexts with similar clinical results. More specifically, our methodology meets the demands of an ART clinic, where reduced timing and work quality must coexist. It is fast, cheap, easy to learn, highly repeatable, and results in pregnancy rates are comparable to the most innovative methods.

## Figures and Tables

**Table 1:** Advantages and disadvantages of sperm cells’ selection techniques.

Procedures	Advantages	Disadvantages
**Swim-up**	▪ Simple, fast and economical. ▪ Isolates motile and morphologically normal spermatozoa [19] ▪ Non-invasive, reduced ROS production and non-fragmented DNA [20] ▪ Reduction proportion of spermatozoa with chromosomal defects [20] ▪ Can be performed after density gradient centrifugation to improve the quality of the recovered spermatozoa [21,22]	▪ Reduced number of recovered spermatozoa [23] ▪ Poor efficiency in the case of high volumes [24] ▪ small ROS production [20]
**Centrifugation on density gradient**	▪ Easy [25] ▪ Isolates a large number of motile and morphologically normal cells [25] ▪ Simple to standardize and adapt in clinical scenarios [26] ▪ Can be conducted in conjunction with swim-up to improve the quality of the recovered spermatozoa [21; 22]	▪ Poor efficiency in case of high viscosity [24] ▪ Toxic effect of Percoll [27] ▪ High ROS production ▪ Overloading the sample can cause the aggregation of sperm cells to other cells [24]
**HOST**	▪ Simple and economical [28] ▪ Evaluation chromatin integrity without damaging the sperm cells [29] ▪ Selects immotile but usable spermatozoa for ICSI based on swelling of the sperm cell tail in a hypo-osmotic environment [30]	▪ Low fertilization rate when incubation in hypo-osmotic solution last longer than 30′ [31] ▪ Poor efficiency in case of a small volume of semen [32]
**Polarization microscopy**	▪ Allows the identification of nuclear structures and the state of the acrosome [33] ▪ The birefringence pattern correlates with sperm cells parameters [34]	▪ Expansive ▪ Long lead times ▪ Requires experienced operators
**LAISS**	▪ Considered a safe method [35] ▪ Selection of immotile spermatozoa but viable for ICSI based on the curling of the flagellum if hit by a laser beam [36] ▪ Recommended for primary ciliary dyskinesia [37] or Kartagener’s syndrome [38]	▪ Expansive ▪ ROS production with high laser doses [39] ▪ Rupture of the plasma membrane with high laser doses [40]
**MACS**	▪ Sperm cell recovery with high motility-normal morphology [41,42] ▪ Sperm cell selection with reduced DNA fragmentation [43,44] ▪ High-quality sperm cell retrieved in combination with SU or DGC [45]	▪ Literature incomplete on the percentage live birth ▪ No discrimination on type of motility
**PICSI and selection with hyaluronic acid**	▪ Hyaluronic acid receptors are expressed only on mature sperm cells [46,47] ▪ Recovery of spermatozoa with reduced aneuploidy [48]	▪ Contradictory results for ART outcomes [49]
**Zeta potential**	▪ Selection of mature spermatozoa based on the negative charge of the plasma membrane [50] ▪ Normal morphology & high percentage DNA integrity [51,52] ▪ It improves the fertilization rate, the percentage of top-quality embryos and of pregnancy when compared to DGC [53]	▪ Potential bias in the selection of spermatozoa containing the X chromosome [50] ▪ There is no association between Z potential and motility, requiring a priori use of other techniques [54,55] ▪ Only one RCT [53]
**IMSI**	▪ Selection based on the observation of the ultra-cellular structure [56] ▪ Benefits in cases of repeated fertilization failures [57]	▪ Expensive ▪ Long lead times ▪ Requires experienced operators ▪ Contradictory results for ART outcomes [58,59]
**Microfluidic separation**	▪ Selection based on morphology and motility [60] ▪ Damage caused by centrifugation is eliminated ▪ Reduced DNA/fragmentation compared to classical methods [61] ▪ Direct use of the sample with automation and scalability	▪ Low volume of yield ▪ High cost ▪ Still not standardized
**Horizontal** **Sperm migration**	▪ Easy, fast and cheap [62] ▪ ROS reduction [62] ▪ Mismatch reduction [62] ▪ Reduced potential for bacterial contamination [62]	▪ Poor applicability to low concentration samples [62] ▪ Results still insufficient for standardization in ART [63]

## Data Availability

Not applicable.
